# Geographic differences in overweight and obesity prevalence in Peruvian children, 2010–2015

**DOI:** 10.1186/s12889-018-5259-2

**Published:** 2018-03-14

**Authors:** J. Smith Torres-Roman, Diego Urrunaga-Pastor, Jose L. Avilez, Luis M. Helguero-Santin, German Malaga

**Affiliations:** 1 Faculty of Medicine, Universidad Nacional San Luis Gonzaga, Ica, Peru; 2 Sociedad Científica de Estudiantes de Medicina, Universidad de San Martín de Porres, Lima, Peru; 3 Faculty of Mathematics, University of Waterloo, Waterloo, Canada; 4 Faculty of Medicine, Universidad Nacional de Piura, Piura, Peru; 5 School of Medicine, Universidad Peruana Cayetano Heredia, Lima, Peru

**Keywords:** Obesity, Overweight, Children, Spatial clustering, GIS, Peru

## Abstract

**Background:**

Childhood obesity is emerging as a major public health problem worldwide. To date, most studies of obesity and overweight in Peru are focused on adults, with few of them involving children, a population at a critical stage of development. The trend in overweight and obesity prevalence in Peruvian children under the age of five has not yet been determined. Thus, the objective of the present study is to evaluate the prevalence rates of overweight and obesity between 2010 and 2015 in children under the age of five, stratified by geographical areas in Peru.

**Methods:**

Data were obtained from the Nutritional Status Information System of Peru. The total number of children evaluated was 14,155,914. For the Geographic Information Systems (GIS), the program Geo Da 1.8® was used to ascertain the spatial distribution of prevalence rates and was mapped for children under five. To assess the degree of spatial dependence, exploratory spatial data analysis was performed using the Moran’s I statistic and was assessed with the Local Indicators of Spatial Association (LISA) analysis to identify geographic concentrations of high and low of obesity and overweight levels.

**Results:**

Between 2010 and 2015, the national prevalence of childhood overweight and obesity ranged from 6.2%- 6.8% and 1.5%–2.7%, respectively. The highest prevalence of childhood overweight and obesity was found in 2014 and 2013, respectively. During these years, we observed that obesity decreased, but overweight remained stable. The highest prevalence of overweight and obesity was found in the departments located on the coast. Significant positive spatial autocorrelation was found for both overweight and obesity. The departments with the highest prevalence of overweight and obesity were concentrated in Lima, Callao, Ica, Moquegua and Tacna. The lowest were found in Loreto, Cusco and San Martin.

**Conclusion:**

The decrease in obesity and the stabilisation of overweight are positive results for the Peruvian childhood. However, in comparison with other Latin American countries, Peru still lags in obesity prevention.

## Background

Economic development, industrialisation, and rapid urbanisation in developing countries have led to the adoption of unhealthy physical, dietary, and lifestyle behaviours, which have, in turn, increased the prevalence of obesity in adults and children [1–4]. For 2013, low- and middle-income countries (LMICs) reported that 42 million children under five had overweight or obesity; this number is expected to surpass 60 million by 2020 [5, 6]. This leaves no doubt that children are one of the populations most affected by overweight and obesity, particularly given these diseases' status as major global public health threats [1, 2, 7, 8].

Latin America is no stranger to this reality. In 2010, approximately 7% of all children under five were overweight or obese, according to the World Health Organisation’s (WHO) standards [2]. Likewise, a 2013 systematic review reported that one in every four or five children or adolescents had overweight or obesity in the region [9]. Furthermore, between 20% and 25% of people under 19-years-old in Latin America is overweight or obese. Amongst Latin American countries, Chile has the greatest prevalence of overweight and obesity, followed by Mexico and Peru [9].

In Peru, the rapid urbanisation has created an “obesogenic” environment, mainly in urban areas which report greater caloric intakes [10] and higher physical inactivity rates [11, 12]. Loret De Mola et al., using the Peruvian National Demographic and Family Health Surveys (DHS) between 1996 and 2011 found that prevalence of overweight and obesity children in urban areas had decreased; however, this trend seems to continue until 2005, since the prevalence of overweight stabilised at around 12% [13].

In Peru, the variation over the last 5 years has not yet been reported in terms of the prevalence of overweight and childhood obesity in Peruvian geographical regions and departments [14]. For this reason, we carried out an analysis of the nutritional status of Peruvian children under 5 years old who attended health centres through the country to determine the prevalence of overweight and obesity between 2010 and 2015 across the different Peruvian geographical areas.

## Methods

### Source of information

A secondary data analysis from the “Nutritional Status Information System database” [15] of the National Institute of Statistics and Informatics of Peru through the National Centre for Food and Nutrition was carried out. The database, registered the information of the nutritional status of under-five years old children who attended public health establishments in Peru between 2010 and 2015, and using the WHO standards for classifying overweight and obesity in childhood as the reference [16].

### Setting and population

According to the geopolitical division, 25 departments, called political regions, constitute Peru distributed in three natural regions: coast, highlands, and rainforest. In each department, there exists a Regional Health Office from Peruvian Ministry of Health. Our work included the 25 departments of the Peruvian territory. Likewise, we divided the country into its three natural regions: coast, highlands, rainforest, and Lima (the capital of Peru, located on the coast). Lima was not included in the coast analysis and was assessed as a separate region as it contains a third of the country population [17] and thus provides the largest number of cases in the study. These sites display a wide array of geographical diversity: the coast has experienced significant and unplanned population growth in relatively urbanised, low altitude settings; the highlands are a high-altitude area, with both urban and rural settlements spanning the Andes range; the rainforest is the largest region in Peru and spans throughout the Amazon rainforest; and Lima, the capital city, is located on the coast and is a highly urbanised with ten million inhabitants.

In total, 14,155,914 children were evaluated during the 6 years of analysis. These were divided into 2,346,373, 2,379,679, 2,359,065, 2,400,384, 2,321,387, 2,349,026 infants in 2010, 2011, 2012, 2013, 2014, and 2015, respectively. The population of children < 5 years included in our study represented between 79.31%–82.08% of the total national population of children under 5 years [18].

### Variables and measurements

The entire population of 14,155,914 children under 5 years included in our study were evaluated by weight and height. The data was collected daily at the primary health care centers [19]. Anthropometric measurements were performed according to the internationally accepted methodology, included in the guide developed by the National Centre for Food and Nutrition [20], using calibrated digital balances with an accuracy of ±100 g and measuring rods made according to the technical specifications of this organisation [16]. The cases of overweight and obesity were classified using WHO definitions, which consider the existence of overweight when the weight-per-unit-height exceeds the median growth pattern by more than two standard deviations (SD), and three SD for the case of obesity [21].

### Statistical analysis

We used Microsoft Excel 2013 for the statistical descriptive analysis. We calculated the prevalence of overweight and obesity for each of the 25 departments of Peru in the study period. We calculated the prevalence of overweight and obesity in each year taking as a numerator the number of cases of overweight or obesity present in each year, and as a denominator the total number of children evaluated respectively in each year.

### Spatial analysis

For the Geographic Information Systems the program Geo Da 1.8® was used to determine the spatial analysis [22]. The spatial analysis was performed using the Moran’s I statistic and the Local Indicator of Spatial Association (LISA).

The map results in a spatial typology consisting of five categories of health regions: (i) ‘high–high’ (positive autocorrelation), (ii) ‘low–high’ (negative autocorrelation), (iii) ‘low–low’ (positive autocorrelation), (iv) ‘high–low’ (negative spatial autocorrelation), and (v) ‘not significant’ indicates that there is no spatial autocorrelation.

The value of the Moran index varies between − 1 and + 1, where negative values indicate a spatial conglomerate of territorial units with different values of analysis and positive values indicate a spatial conglomerate of territorial units with similar values of analysis.

## Results

Table 1 presents the Moran’s I statistic with significance *p*-values. The results indicated that there is significant positive spatial autocorrelation in the data. The level of differed autocorrelation differed among overweight and obesity. The level of spatial autocorrelation across departments for overweight prevalence rates was significant 0.4 with *p* < 0.05 in the period study. Conversely, the spatial autocorrelation for the obesity prevalence rates was 0.2854, 0.3112, 0.3318, 03238, 0.3293, 0.2956 in the years 2010, 2011, 2012, 2013, 2014, 2015, respectively. However, all these previously mentioned were not significant.

**Table 1:** Spatial autocorrelation of prevalence overweight and obesity rates in Peru in 2010–2015

	Moran ’s I	*p*-value
Overweight		
2010	0.4069	< 0.05
2011	0.4053	< 0.05
2012	0.4230	< 0.05
2013	0.4131	< 0.05
2014	0.4009	< 0.05
2015	0.3840	< 0.05
Obesity		
2010	0.2854	0.015
2011	0.3112	0.013
2012	0.3318	0.011
2013	0.3238	0.008
2014	0.3293	0.006
2015	0.2956	0.014

**Fig. 1:**
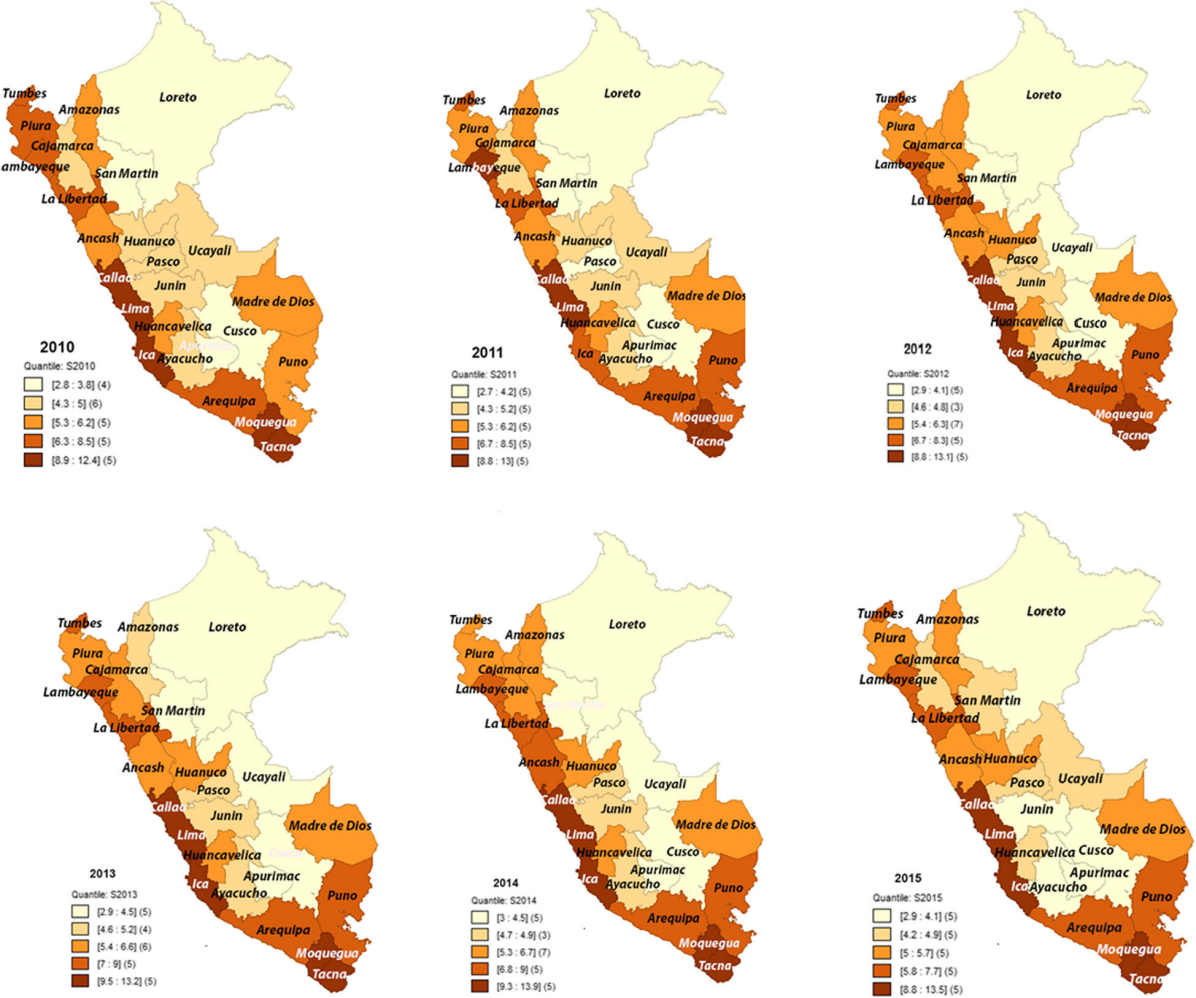
Spatial distribution of prevalence rates of overweight in children under 5 in Peru, 2010–2015

Lima, Callao, Ica, Moquegua, and Tacna, five departments along the coast, had the highest prevalence of overweight with a range of 8.8%–13.9%. Conversely, the lowest prevalence rates of overweight were found in Loreto, Cusco, San Martín, and Apurimac with a range of 0.8%–4.5%, of which two are in the highlands and two in the rainforest. See Fig. 1.

**Fig. 2:**
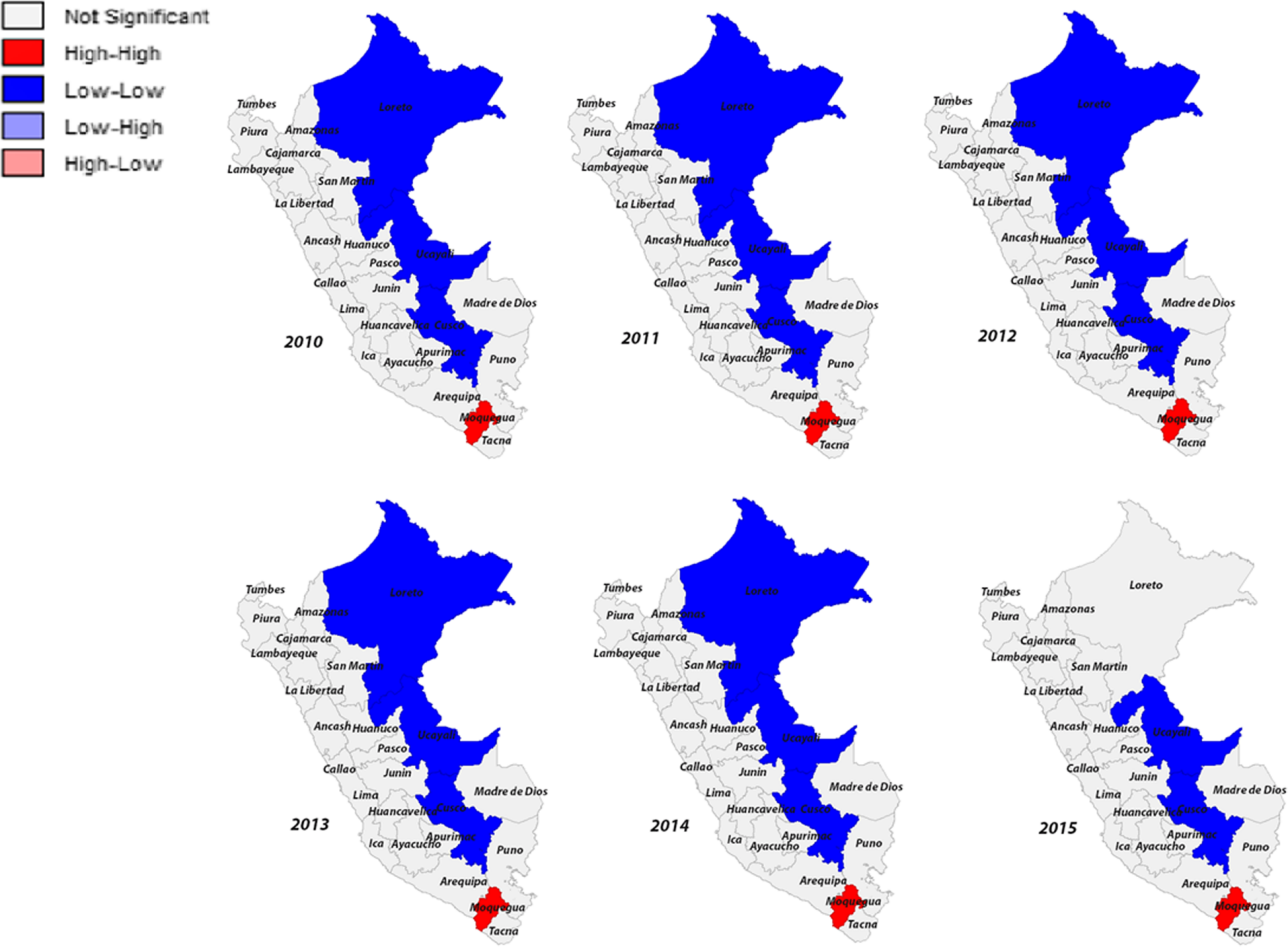
LISA cluster maps for prevalence rates of overweight in 2010–2015 by departments in Peru

For Fig. 2, the results of the spatial analysis of overweight indicated that there was a autocorrelation between departments with a Moran’s I that oscillates around 0.4 across the 6 years of study. The LISA analysis indicated that the spatial autocorrelation was positive by presenting near values. Similarly, between the years 2010–2014, the “Low-Low” departments were Loreto, Ucayali, and Cusco. The “High-High” department in the study period was Moquegua.

**Fig. 3:**
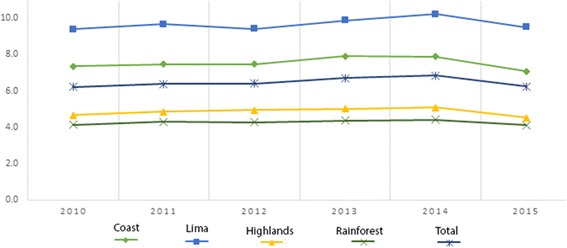
Prevalence of overweight in children under 5 by natural region in Peru, 2010–2015

For the years 2010–2015, the national prevalence of childhood overweight fluctuated between 6.2% and 6.8%, reaching its highest prevalence in 2014 with 6.8%. Furthermore, for 2010, the prevalence of overweight in the coast was 7.4%, reaching 7.9% in 2013 and decreasing to 7.1% in 2015. For Lima, the prevalence was 9.4% in 2010, reaching its highest prevalence in 2014 with 10.2%. On the other hand, in the highlands, the prevalence was 4.7% in 2010, then increased to 5.1% in 2014 and decreased by 0.7%, until finally reaching 4.5% in 2015. Finally, for rainforest, the prevalence in 2010 was 4.1, reaching 4.4% in 2014. See Fig. 3.

**Fig. 4:**
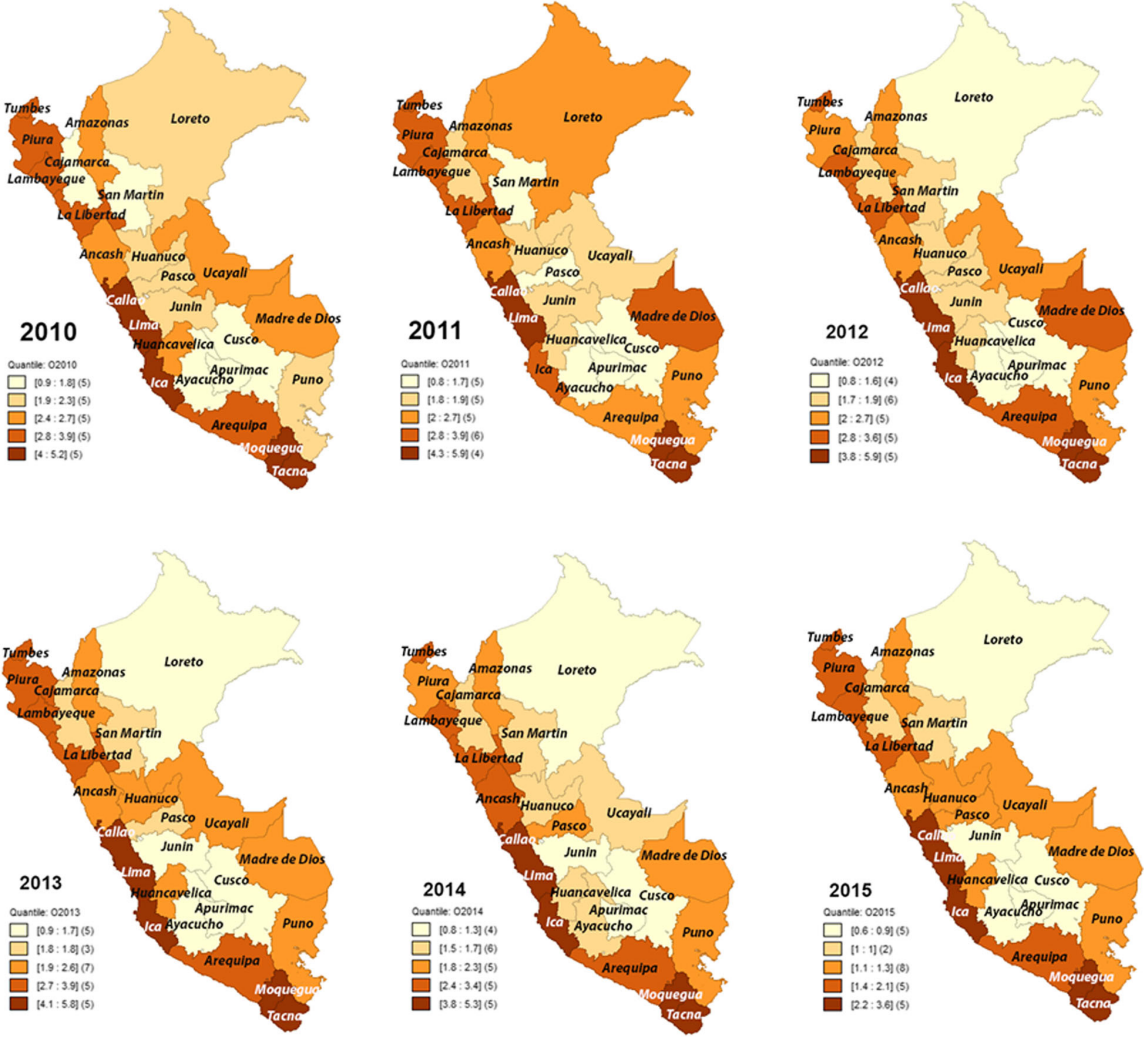
Spatial distribution of prevalence rates of obesity in children under 5 in Peru, 2010–2015

In the Fig. 4 the departments with the highest prevalence of obesity were Lima, Callao, Ica, Moquegua and Tacna with a range of 2.2%–5.9%. The lowest prevalence rates of obesity were found in Loreto, San Martín, Cusco, Ayacucho and Apurimac with a range of 0.6%–1.8%.

**Fig. 5:**
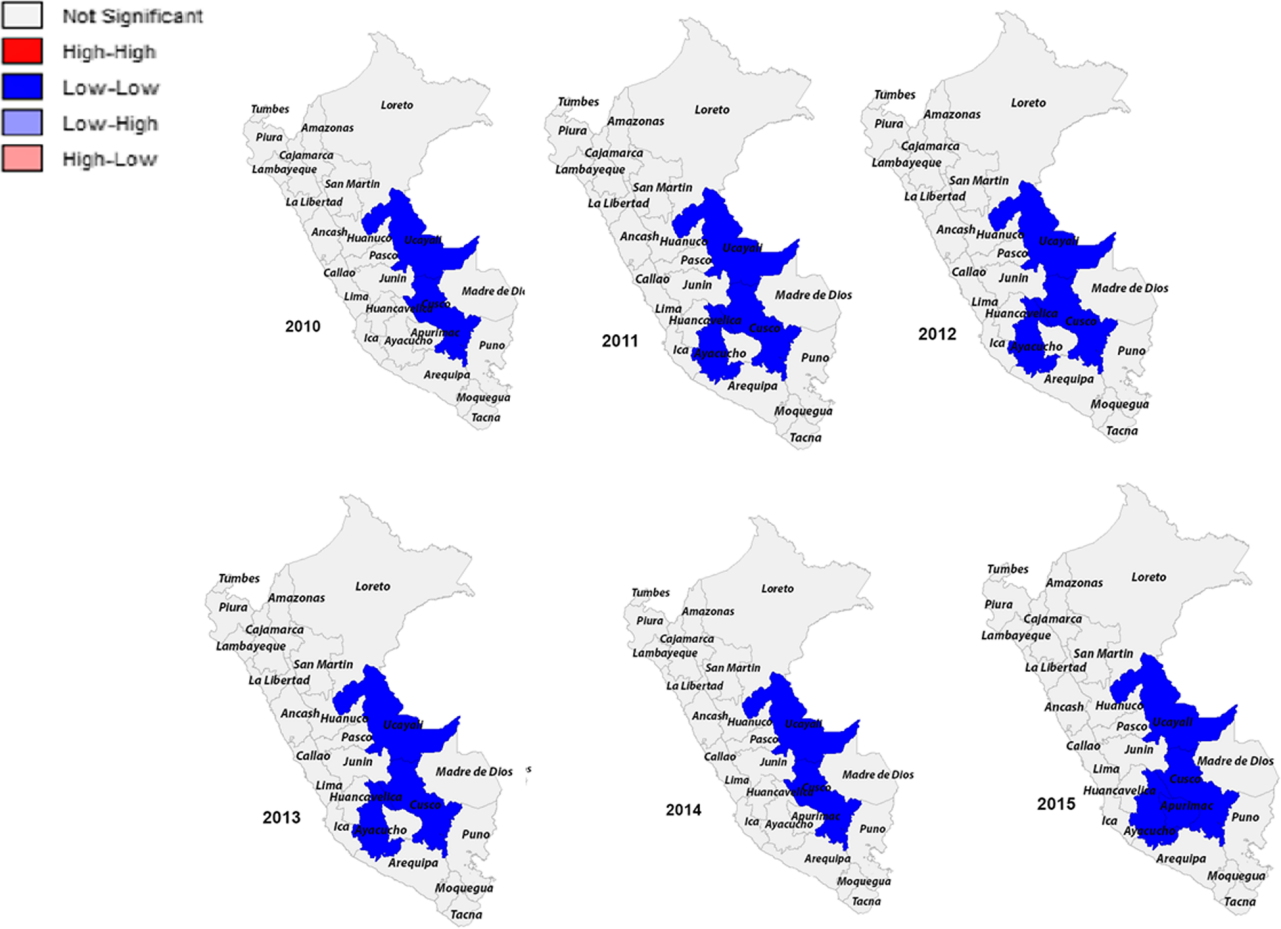
LISA cluster maps for prevalence rates of obesity in 2010–2015 by departments in Peru

For Fig. 5, presents a LISA map of significant high- and low-obesity departments clusters within each region based on a random permutation procedure. The LISA analysis indicated spatial autocorrelation was positive by presenting near values. In 2010 and 2014, the “Low-Low” departments were Ucayali and Cusco. In 2011, 2012 and 2013, the “Low-Low” departments were Ucayali, Cusco, and Ayacucho. Finally, in 2015 the “Low-Low” departments were Ucayali, Cusco, Ayacucho and Apurimac.

**Fig. 6:**
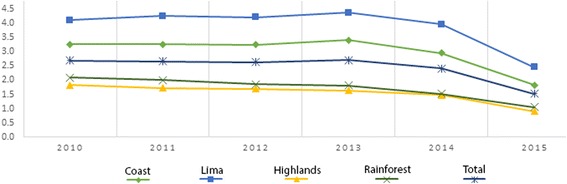
Prevalence of obesity in children under 5 by natural region in Peru, 2010–2015

In 2010, the national prevalence of obesity was 2.69%, following a variable prevalence trend over time, reaching its highest point in 2013 with 2.72% and decreasing on average 0.6% annually, reaching its lowest point in 2015 with a prevalence of obesity of 1.5% which represented that year a total of 35,669 children under 5 years. For the year 2010, the prevalence of obesity among children under 5 years on the coast was 3.3%, reaching a maximum prevalence of 3.4% in 2013. In addition, for Lima, the prevalence was 4.1% in 2010, and its highest prevalence was observed in 2013. In the highlands, the prevalence was 1.8% in 2010, then it began to decrease, on average, by 0.2% every year until reaching a prevalence of 0.90% in 2015. Finally, the prevalence of obesity in the rainforest was 2.1% in 2010, starting an average decrease of 0.2% annually until reaching a prevalence of 1.1% in 2015. See Fig. 6.

## Discussion

The study suggests a marked variability of overweight and obesity prevalence in Peruvian children when compared by departments, which had been demonstrated by the spatial heterogeneity showed in the positive spatial autocorrelation identified by Moran’s I for children as well as the significant local clusters identified by LISA [23] and reaffirmed the necessity and importance of developing both nationwide and region-specific prevention programmes and health-related policies.

The spatial distribution between 2010 and 2015, showed no final changes in overweight. During the period described by the National Household Survey (2009–2010) a 6.4% of children under 5 years were overweight [24]. In addition, results reported by Urke et al., using cross-sectional data from the DHS in Peru, data collected in five time intervals (1991–1992, 1996, 2000, 2007–2008, and 2011), did not find any general change in the prevalence of overweight in children under 5 years [25]. Thus, our study confirms that overweight statistics has remained stable in the last 5 years of study.

In this study, we found that different patterns of prevalence of overweight and obesity are higher in Lima and the coast than compared to the highlands and the rainforest [24]. These differences had been described before. For example, it was found in previous studies that, between 2007 and 2010, the highest prevalence of overweight and obesity in Metropolitan Lima was 10.1%, while in the rainforest only 2.6% in children under 5 years [26] suffered from this problem. Conversely, Tarqui et al. [27] found that, during the period 2007–2011, the coast displayed higher prevalence rates on average than Metropolitan Lima (7.6% vs 7.3%). In other countries, increased levels of overweight and obesity have been linked to urbanisation [28, 29], which is related to an increase in poor eating habits [30]. Furthermore, it has been described that people living in the lowlands are more likely to develop obesity than those who live in the highlands [31]. This would be a possible explanation for the differences in the prevalence of overweight and obesity found in the coast and Lima located in lowlands.

This study indicated that the departments of Moquegua, Tacna, Lima, Callao, and Ica had highest prevalence rates of overweight and obesity according to the spatial distribution. This problem may be attributed to the non-participation of the Ministry of Health of the Peru, which has not yet implemented Law N° 30021, entitled “Promotion of Healthy Food for Children and Adolescents” [32, 33]. The law’s primary objective is to promote physical activities and implement kiosks and healthy eating places in education institutions, with the fundamental objective of reducing overweight and obesity in Peruvian children. For this reason, Peru needs continued focus on body-mass index surveillance and identification, implementation, and evaluation of obesity prevention policies mainly in the departments with a high prevalence of obesity and overweight in Peru. Likewise, it is important to provide medical care and advice to overweight and obese children, and their parents, in the mentioned departments, to avoid the appearance of metabolic diseases at an early age or adverse consequences of premature death and morbidity in adulthood.

We recommend that the Peruvian government implement public policies and prioritise overweight and obesity prevention in each department within its national health policy. These actions are intended to promote healthy lifestyles, such as healthy eating, more physical activity, reduction in sedentarism, and reduction in the use of television and computers in school children [34]. These policies, under the framework of Law N° 30021, should strive to monitor weight loss behaviour in the general population, mainly in children, as well as monitor caloric intake, by studying the composition of diets of children on a regional basis, and act upon the findings of these systems [34]. It is important to focus the interventions in children between 5 and 6 years old, considering the early adiposity rebound, a significant increase in body fat proportion and body mass composition at this age [31]. Prevention in children is critical, as obesity at this age dramatically increases the risk of adult obesity [35] and obesity-related diseases, such as hypertension, diabetes, and some types of cancer [36]. Since children begin attending school at this age, it is crucial that Law N° 30021 and other forms of legislation are properly implemented to contribute to a further decrease in rates of overweight and obesity in each Peruvian department.

## Limitations

The Nutritional Status Information System database lacked demographical variables including sex, religion, income level, and education of parents; furthermore, other strong predictors of obesity, such as culture, knowledge and perception of obesity. However, the information obtained by the National Centre for Food and Nutrition is representative, allowing comparisons the geographic areas of the Peru. The reported results can serve to observe the patterns of departmental, regional and national prevalence of overweight and obesity. In addition, this study was the first to analyse the departments geographically, which allowed us to observe clusters of the high prevalence of overweight and obesity in children under 5 years of age in Peru.

## Conclusion

The findings established in this study confirming marked geographic differences in the prevalence of overweight and obesity in children Peruvians. In addition, the decrease in the prevalence of obesity in some departments and the stabilisation of overweight rates in most departments show us a beacon of hope in the fight against overweight in Peruvian children.

## Abbreviations

DHS: Demographic and Family Health Surveys
GIS: Geographic Information Systems
LISA: Local Indicators of Spatial Association
LMICs: Low- and middle-income countries
SD: Standard deviations
WHO: World Health Organisation’s
